# Association of breastfeeding and postmenopausal osteoporosis in Chinese women: a community-based retrospective study

**DOI:** 10.1186/s12905-019-0808-0

**Published:** 2019-08-13

**Authors:** Guiming Yan, Yaqi Huang, Hong Cao, Jie Wu, Nan Jiang, Xiaona Cao

**Affiliations:** 1 School of Nursing, Tianjin Medical University, Tianjin, 300070 China; 2 Department of bone medicine of Tianjin Hospital, Tianjin, China; 3 Xiao Bai Lou Community Healthcare Service Center, Heping district, Tianjin, China

**Keywords:** Breastfeeding, Osteoporosis, Postmenopausal, Public health, Community-based participatory research, Retrospective study, Cross-sectional study

## Abstract

**Background:**

Postmenopausal osteoporosis (PMOP) has long been a pervasive public health concern. With the aging Chinese population, the prevention, assessment and management of postmenopausal osteoporosis were particularly important. During the breastfeeding, a large amount of Calcium loss from maternal bone for infants’ growth. However, whether this loss is completely reversible remains controversial. As the relationship between breastfeeding and postmenopausal osteoporosis is different from society to society and is not clear from the literature, the purpose of this study was to determine whether breastfeeding was an independent factor for the development of PMOP based on Chinese postmenopausal population.

**Methods:**

A retrospective cross-sectional investigation was conducted at Tianjin Xiaobailou health Community Healthcare Center between December 2017 and June 2018. Postmenopausal women over the age of 50 who underwent the annual health examination or visited the center to perform bone densitometry as a part of routine screening for disease were recruited. A trained community nurse administered a questionnaire to all participants by face-to-face interview. Participants were questioned about age, BMI, Vitamin D and calcium intake, the history of smoking, drinking and fracture, age of menarche, age of menopause, the number of pregnancy, parity, feeding pattern (breastfeeding, artificial feeding and mixed feeding) and overall breastfeeding duration. BMD measurements were carried out using quantitative ultrasound (QUS) at the bilateral radius.

**Results:**

A total of 202 women who met the inclusive and exclusive criteria were enrolled. Univariate analysis revealed that overall breastfeeding more than 24 months increased the risk of osteoporosis (OR 39.00, 95%CI 2.40–634.65, *p* = 0.010). However, multivariate estimate of the risk of osteoporosis by overall breastfeeding duration suggested that when controlling for age, BMI, the number of pregnancy and parity, the overall breastfeeding duration was not an independent risk factor for postmenopausal osteoporosis (OR 5.22, 95%CI 0.18–147.76, *p* = 0.333). Additionally, age (OR 1.16, 95%CI 1.05–1.29, *p* = 0.003), BMI (OR 1.26, 95%CI 1.04–1.54, *p* = 0.021) and the number of pregnancy (OR 1.80, 95%CI 1.08–2.98, *p* = 0.024) were significant associated with postmenopausal osteoporosis.

**Conclusion:**

Breastfeeding was not associated with postmenopausal osteoporosis, while age, BMI and the number of pregnancy may contribute to increasing risk of postmenopausal osteoporosis in Chinese women.

**Electronic supplementary material:**

The online version of this article (10.1186/s12905-019-0808-0) contains supplementary material, which is available to authorized users.

## Background

Osteoporosis is one of the most common chronic metabolic skeletal disease defined by low bone mass and disruption of bone microarchitecture, with increased risk of bone fragility and fracture. Postmenopausal women were considered to be at high risk of developing osteoporosis, as Estrogen deficiency accelerated bone turnover with net bone loss [1]. Postmenopausal osteoporosis (PMOP) has long been a pervasive public health concern. In China, an approximately 40.1% prevalence of PMOP was reported in a nationwide study [2]. The most important adverse health outcome of osteoporosis contributes to the occurrence of any bone fractures, which reduced the health-related quality of life in all aspects [3]. It was estimated that the residual lifetime risk (RLR) of any fracture for 50-year-old Chinese woman was 37.6% [4]. Therefore, with the aging Chinese population, the prevention, assessment and management of postmenopausal osteoporosis were particularly important. Pregnancy and lactation period were considered to affect bone metabolism and calcium homeostasis. In this period, increased intestinal calcium absorption and breast milk secretion cause the calcium loss in maternal skeleton, but the bone loss could restore within 6–12 months after weaning [5]. However, it is unclear whether the bone loss is completely compensated. It has been reported that prolonged total breastfeeding time was significantly associated with decreased bone mineral density (BMD) [6, 7]. But as well known, PMOP was multifactorial, and its related factors also include age, body mass index (BMI), smoking, alcoholic, physical activity, extended calcium and Vitamin D intake, pregnancy interval, the age of pregnancy, multiple births, the age of menarche and menopause status [8–11]. Studies on factors correlated to breastfeeding, as independent risk factors for PMOP remain controversial [12]. Some studies suggested that the history of breastfeeding can significantly increased the risk of PMOP [13], while some studies showed that prolonged breastfeeding duration was an independent risk factor for the development of PMOP rather than breastfeeding itself [14, 15]. Conversely, some studies suggested that the history of breastfeeding may increase the bone mass density, which significantly decreased the incidence of PMOP [16], whereas other studies demonstrated a non-significant relationship between breast-feeding and PMOP [9, 17].

As the relationship between breastfeeding and PMOP is different from society to society and is not clear from the literature above, the purpose of this retrospective cross-sectional study was to determine whether breastfeeding and its total breastfeeding duration was an independent factor for the development of PMOP based on Chinese postmenopausal population.

## Method

### Study design and population

A retrospective cross-sectional investigation was conducted at Tianjin Xiaobailou health Community Healthcare Center between December 2017 and June 2018. Postmenopausal women over the age of 50 who underwent the annual health examination or visited the center to perform bone densitometry as a part of routine screening for disease were included. Women were excluded if they had a history of osteoporosis treatment (e.g. osteoporosis medication, hormone replacement therapy), menopausal hormone use, oophorectomy and oral contraceptive or any drugs that have definitely effect on bone metabolism. To exclude the secondary causes of osteoporosis, women with metabolism disorders or autoimmune disease (e.g. thyroid disease, chronic or severe liver disease, chronic malnutrition, chronic renal failure, inflammatory rheumatic disease, malignancy) were not included in this study. Some longitudinal studies showed that nulliparity was a highly risk factor of decreased BMD rather than non-breastfeeding [6], therefore, women who had never given birth were removed from the sample. Additionally, participants with any memory or cognitive impairment were excluded to avoid the recall bias, and incomplete questionnaires were also removed.

A trained community nurse administered a questionnaire to all participants by face-to-face interview. An informed consent was obtained prior to the investigation. The questionnaire consisted of PMOP associated demographic information and reproductive factors (Additional file 1). The questions regarding to demographic information including age, height, weight, BMI, history of smoking, drinking and fracture, Vitamin D and calcium intake. Body weight and height were measured by trained nurses, and BMI (kg/m^2^) was calculated. Reproductive factors including age of menarche, age of menopause, the number of pregnancy, parity, feeding pattern (breastfeeding, artificial feeding and mixed feeding) and overall breastfeeding duration. BMD measurements were carried out using quantitative ultrasound (QUS) at the bilateral radius (OSTEO KJ3000S+) to divide individuals into non-osteoporosis group and osteoporosis group. Osteoporosis was defined as a T score of − 2.5 or lower emerging from the results of the QUS obtained from one side of the bilateral radius.

### Statistical analyses

All data were analyzed using the survey procedure of IBM SPSS version 22.0 (SPSS, Chicago, IL, USA). Participants’ characteristics were presented as means (±Standard Deviations) for continuous variables, and counts and percentages for categorical variables. Kolmogorov-Smirnov test was performed to assess the normal distributions of continuous variables. All the data were analyzed after grouping participants according to the presence or absence of postmenopausal osteoporosis. Baseline characteristics were compared via Independent Samples *t* test, Chi-squared test or Fischer’s exact tests. The Mann-Whitney U test was conducted for abnormally distributed variables. Cumulative breastfeeding duration as continuous variable and categorical variable were analyzed respectively to examine the association between breastfeeding and postmenopausal osteoporosis. For categorical variable, breastfeeding duration data were grouped into four quartiles: Never, 1-12 months, 12–24 months, and > 24 month. Subsequently, univariate and multivariate logistic regression were carried out to determine the effect of breastfeeding duration and other covariates that identified significant in univariate analysis. Unadjusted and adjusted odd ratio (OR) values with 95% confidence interval (95%CI) were calculated. All statistic tests were two-tailed, with *p*-value < 0.05 being assumed statistically significant.

## Result

### Demographic characteristics

A total of 202 women who met the inclusive and exclusive criteria with a mean age of 69.42 ± 6.48 were enrolled. Mean BMI was 24.11 ± 1.86. Of the 202 women included in the study, 10 individual reported a history of smoking, and 2 individual admitted to take alcohol during their life time. Only 4 women reported a history of fracture. One hundred and thirty-two (65.3%) women’s daily calcium intake less than 500 mg, and 138 (68.3%) women took less than 400 IU Vitamin D per day. Of the participants, 150 participants (74.3%) were diagnosed with postmenopausal osteoporosis according to the BMD value and 52 participants (25.7%) were with normal BMD. When comparing the demographic characteristic in the non-osteoporosis group and osteoporosis group, the mean age of participants in osteoporosis group was significant older than those of in non-osteoporosis group (*p* < 0.001). Additionally, the BMI of participants in osteoporosis group was significantly higher (*p* = 0.033). There was no other significant difference detected between the two groups (Table 1).

**Table 1:** Demographic characteristics of the study population

Characteristic	All women (*n* = 202)	Non-osteoporosis (*n* = 52)	Osteoporosis (*n* = 150)	*p-*value*
Age (year)	69.42 ± 6.48	65.29 ± 3.877	70.85 ± 6.60	**< 0.001**
BMI (kg/m^2^)	24.11 ± 1.86	23.65 ± 1.76	24.27 ± 1.87	**0.033**
Height (cm)	159.78 ± 4.41	161.48 ± 4.25	159.19 ± 4.35	**0.001**
Weight (kg)	61.64 ± 6.28	61.74 ± 6.03	61.60 ± 6.38	0.890
Smoking,*n* (%)				
Yes	10 (5.0%)	0 (0.0%)	10 (6.7%)	0.056
No	192 (95.0%)	52 (100%)	140 (93.3%)	
Alcohol, *n* (%)				
Yes	2 (1.0%)	0 (0.0%)	2 (1.3%)	0.403
No	200 (99.0%)	52 (100%)	148 (98.7%)	
Fracture, *n* (%)				
Yes	4 (2.0%)	1 (1.9%)	3 (2.0%)	0.973
No	198 (98.0%)	51 (98.1%)	147 (98%)	
Calcium intake per day *n* (%)				
≤ 500 mg	132 (65.3%)	34 (65.4%)	98 (65.3%)	0.995
> 500 mg	70 (34.7%)	18 (34.6%)	52 (34.7%)	
Vitamin D intake per day *n* (%)				
≤ 400 IU	138 (68.3%)	36 (69.2%)	102 (68.0%)	0.869
> 400 IU	64 (31.7%)	16 (30.8%)	48 (32.0%)	

*BMI* body mass index.

*p-value are calculate using Chi-square test for categorical variables, t-test for continuous variables

*p*<0.05 were considered significant. Entries in boldface are significant

### Reproductive features

Comparisons of reproductive features between the groups are shown in Table 2. The number of pregnancy was 3.33 ± 0.81 in non-osteoporosis group and 4.20 ± 1.14 in osteoporosis group, which indicated a statistically significant difference between the groups. A significant difference was also identified in parity. The women in osteoporosis group had more deliveries than those in non-osteoporosis group (*p* < 0.001). We found that osteoporosis group had prolonged breastfeeding duration in terms of both continuous variable and categorical variable (*p* = 0.002). However, the menarche age, menopause age and feeding pattern in non-osteoporosis group were not significant as compared to osteoporosis group (*p* = 0.083, *p* = 0.072, *p* = 0.362, respectively).

**Table 2:** Reproductive features in study population

Characteristic	All women (*n* = 202)	Non-osteoporosis (*n* = 52)	Osteoporosis (*n* = 150)	*p-*value*
Menarche age	12.94 ± 0.95	12.73 ± 0.99	13.01 ± 0.93	0.083
Menopause age	50.76 ± 2.62	50.21 ± 2.50	50.95 ± 2.64	0.072
Number of pregnancy	3.98 ± 1.13	3.33 ± 0.81	4.20 ± 1.14	**< 0.001**
Parity	1.77 ± 0.97	1.31 ± 0.64	1.93 ± 1.02	**< 0.001**
Feeding pattern, *n* (%)				
Breastfeeding	186 (92.1%)	49 (94.3%)	137 (91.3%)	0.362
Artificial feeding	4 (2.0%)	2 (3.8%)	2 (1.3%)	
Mixed feeding	12 (5.9%)	1 (1.9%)	11 (7.4%)	
Overall breastfeeding duration	21.68 ± 12.78	16.88 ± 9.86	23.35 ± 13.28	**0.002**
Overall breastfeeding duration, n (%)				
Never	4 (2.0%)	2 (3.8%)	2 (1.3%)	**0.002**
1–12 months	84 (41.6%)	26 (50.0%)	58 (38.7%)	
12–24 months	74 (36.6%)	23 (44.3%)	51 (34.0%)	
> 24 month	40 (19.8%)	1 (1.9%)	39 (26.0%)	

*p-value are calculate using Chi-square test for categorical variables, *t*-test for continuous variables

*p*<0.05 were considered significant. Entries in boldface are significant

### Breastfeeding and postmenopausal osteoporosis

The results of logistic analysis are presented in Table 3. Univariate analysis revealed that overall breastfeeding more than 24 months increased the risk of osteoporosis (OR 39.00, 95%CI 2.40–634.65, *p* = 0.010). However, multivariate estimate of the risk of osteoporosis by overall breastfeeding duration suggested that when controlling for age, BMI, the number of pregnancy and parity, the overall breastfeeding duration was not an independent risk factor for postmenopausal osteoporosis (OR 5.22, 95%CI 0.18–147.76, *p* = 0.333). Additionally, age (OR 1.16, 95%CI 1.05–1.29, *p* = 0.003), BMI (OR 1.26, 95%CI 1.04–1.54, *p* = 0.021) and the number of pregnancy (OR 1.80, 95%CI 1.08–2.98, *p* = 0.024) were significant associated with postmenopausal osteoporosis.

**Table 3:** Logistic regression analysis of risk factors for postmenopausal osteoporosis

	Unadjusted OR (95% CI)	*p*-value	Adjusted OR (95% CI)	*p*-value
Age	1.19 (1.11–1.28)	**< 0.001**	1.16 (1.05–1.29)	**0.003**
BMI	1.21 (1.01–1.44)	**0.039**	1.26 (1.04–1.54)	**0.021**
The number of pregnancy	2.50 (1.67–3.75)	**< 0.001**	1.80 (1.08–2.98)	**0.024**
parity	2.61 (1.58–4.32)	**< 0.001**	0.78 (0.29–2.10)	0.625
Overall breastfeeding duration				
Never	1 (reference)		1 (reference)	
1–12 months	2.23 (0.30–16.71)	0.435	2.51 (0.26–23.90)	0.424
12–24 months	2.22 (0.29–16.73)	0.440	1.26 (0.13–12.30)	0.840
> 24 months	39.00 (2.40–634.65)	**0.010**	5.22 (0.18–147.76)	0.333

Abbreviations: *OR* Odds Ratio, *BMI* body mass index

*p*<0.05 were considered significant. Entries in boldface are significant

## Discussion

The present study examined the association of breastfeeding and the development of PMOP in a population of Chinese postmenopausal women undergoing routine BMD test in a community healthcare center. The main finding of study was that longer breastfeeding duration was not significantly associated with an increased risk for postmenopausal osteoporosis (OR 5.22, 95%CI 0.18–147.76, *p* = 0.333) when adjusting for the age, BMI, the number of pregnancy and parity. We also observed that advanced age, higher BMI, and more pregnancy were independent risk factors related to PMOP.

The association between PMOP and the total duration of breastfeeding were seen to be complex and differ from society to society. In Korea, a nationwide survey showed that breastfeeding more than 37 months was significantly associated with low BDM and high risk of PMOP [7, 14], similar result was also found in Israel [6] and Mexico [8]. Moreover, an Italy study suggested that long periods of breastfeeding significantly increased the risk of osteoporotic fracture after menopause [18]. In contrast, another study in US suggested that the history of breastfeeding protected against the development of PMOP, especially in women aged 49–54 [19], while a research conducted with 93,676 postmenopausal women in US held different point of view. It was reported that breastfeeding more than 1 month was largely unrelated to decreased BMD and any osteoporotic fracture, additionally, breastfeeding more than 1 month could decreased the risk of osteoporotic fracture comparing with never breastfeeding [20]. In the study of Sri Lanka, prolonged breastfeeding had no detrimental effect on BMD in postmenopausal women [21]. Similarly, in a latest study in Turkey, no statistically significantly difference was examined among the groups (normal, osteopenia, and osteoporosis) in respect of total breastfeeding time, but whether breastfeeding was a risk factor for osteoporosis compared to other types of feeding was not involved [17]. These discrepancies above may have been due to the differences in study design and study population. In current study, we supported that breastfeeding and its duration did not associate with the development of PMOP comparing with non-breastfeeding and mixed feeding. The prolonged breastfeeding was not an independent risk factor for PMOP in the population of Chinese postmenopausal women.

There were several evidence support our findings. First, during the pregnancy and lactation, the mechanism of bone calcium protection may be activated. On the one hand, increased intestinal absorption and renal conservation of calcium compensated for the loss of calcium into the breast milk to reduce the extent of bone loss [22]. On the other hand, the secretion of parathyroid hormone related protein (PTHrP) counterbalances bone loss. PTHrP suppresses parathyroid hormone (PTH) to regulate placental calcium transport and protect maternal skeleton. Besides, prolonged breastfeeding maintains the ovarian in an inactive level. During the breastfeeding, the estrogen level increased, which may keep the balance between bone resorption and formation [5, 12]. Some researchers considered that the ovarian with low activity may contribute to the contraceptive effect of breastfeeding [17]. Actually, the pregnancy interval and the number of pregnancy are independent factors for bone recovery instead of breastfeeding [9]. In our study, we also found that more pregnancies were associated with the risk of PMOP (OR 1.80, 95%CI 1.08–2.98, *p* = 0.024), which confirmed the above hypothesis. Secondly, an animal experiment in rats suggested that breastfeeding had no significant association with PMOP, because pre-reproductive females had higher trabecular bone mass, greater microarchitecture and bone strength than males, which minimized the effect of bone loss on the development of osteoporosis. In addition, when normalized for their body size, post-reproductive females had no skeletal deficits compared to males. This animal experiment showed that females may initially had more trabecular bone than necessary to compensate for possible bone loss due to pregnancy and longer breastfeeding [23], therefore, it helps to explain the result of our study that the prolonged breastfeeding will not increase the risk of PMOP. However, this hypothesis still need to be validated by empirical studies based on larger population.

As breastfeeding and its duration were not risk factors for PMOP, the present study recommends breastfeeding for newborn. It has been reported that breastfeeding had potential benefits to the women’s health [24]. For short-term effect, breastfeeding may decrease the prevalence of postpartum depression [25]. For long-term effect, some studies showed that breastfeeding had protective effect on the prevention of carcinoma, such as breast cancer [26, 27], and ovarian carcinoma [28]. However, the frequency and intensity of breastfeeding implementation in different periods of infants need to be explored in detail, because the process of bone formation during the lactation is still not very clear.

The study is the first to explore the effect of breastfeeding and its duration on PMOP in representative Chinese postmenopausal women based on the community healthcare center. We also considered factors that could affect BMD, such as the age, BMI, smoking, alcohol intake, the history of fracture, calcium and Vitamin D intake, menarche age, menopause age, the number of pregnancy and parity, feeding pattern were included for univariate and multivariate analysis. To minimize the confounding effect of nulliparity on cumulative breastfeeding duration and PMOP, women without the history of pregnancy were excluded from this study. Since the bone mineral are primarily lost to infants during lactation, we hypothesized the amount of breastfeeding can affect the development of PMOP. Although the frequency and duration of daily breastfeeding were not calculated in this study, the feeding pattern and the total breastfeeding duration were considered to indirectly reflect the cumulative amount of breastfeeding, which is the strength of the study.

Limited by facility condition, BMD was measured using the technique of QUS instead of a dual X-ray absorptiometer (DXA). Although QUS has been considered as effective as DXA in determining BMD without using ionizing radiation and non-invasive [29], QUS in community healthcare center failed to measure BMD in multi-site of the body. Indeed, the inconsistent result of the relationship between breastfeeding and PMOP may due to different testing region. In the 2010 Korea National Health and Nutrition Examination Survey, a deleterious effect of prolonged breastfeeding duration on lumbar spine BMD was found, but this conclusion was discrepant in femur neck and total femur [13]. Moreover, animal experiments also showed that the capability of recovery of bone mass may differ from site to site [30, 31]. Therefore, further studies were necessary to explore the effect of breastfeeding on bone mass in which skeletal sites may be irreversibly. Another limitation is because of limited sample size and retrospective cross-sectional study design. It was possible that participants were too old to recall accurate information, which may cause recall bias, and we did not know whether participants had had osteoporosis before menopause. The next step is to conduct a multi-center prospective cohort study with large sample size to confirm the result of our study. Moreover, this study only recorded the amount of additional calcium and vitamins D taken by participants without considering dietary intake. However, it was reported that dietary Calcium may not related to postmenopausal bone loss [32], so this bias had little impact on the results of our study.

## Conclusion

In conclusion, this study indicates that breastfeeding and its duration are not associated with the development of PMOP. Moreover, the prevalence of PMOP was significantly higher in Chinese postmenopausal women with advanced age, higher BMI and more pregnancies. Although the mechanism of bone recovery during lactation is not clear, our findings suggest that an appropriate physical activity and family planning are necessary to control BMI and the number of pregnancy for women bone health.

## Additional file

Additional file 1: Breastfeeding & Postmenopausal Osteoporosis Questionnaire. (DOCX 12 kb)

## Data Availability

The datasets generated and/or analyzed during the current study are not publicly available due to potential for individual and organizational privacy to be compromised. Reasonable requests for parts of the data will be considered by the corresponding author.

## Abbreviations

95%CI: 95% confidence interval
BMD: Bone mineral density
BMI: Body mass index
DXA: Dual X-ray absorption
OR: Odds ratio
PMOP: Postmenopausal osteoporosis
PTH: Parathyroid hormone
PTHrP: Parathyroid hormone related protein
QUS: Quantitative ultrasound
