# Supplementary material for: Association of breastfeeding and postmenopausal osteoporosis in Chinese women: a community-based retrospective study
Source: BMC Womens Health. 2019 Aug 13;19:110. doi: 10.1186/s12905-019-0808-0 (PMC6692954; doi:10.1186/s12905-019-0808-0)
Supplement: Supplementary file 1 — Breastfeeding & Postmenopausal Osteoporosis Questionnaire. (DOCX 12 kb) [file 12905_2019_808_MOESM1_ESM.docx]

Additional file 1

Breastfeeding & Postmenopausal Osteoporosis Questionnaire

1. Age: XX years old

2. Height: XX cm

3. Weight: XX Kg

4. Smoking history: No smoking; Yes: XX year

5. Drinking history: No drinking; Yes: XX year

6. Osteoporosis:

(1) Diagnosis: No Yes (QUS at the bilateral radius)

(2) Fracture History: No Yes: XXX (Where was the fracture)

7. Age of menarche: XX years old

8. Age of menopause: XX years old

9. Times of pregnancy: XX time(s)

10. Parity (times of delivery): XX time(s); Delivery mode: XXX

11. Feeding pattern: breastfeeding, artificial feeding, or mixed feeding

12. Breastfeeding duration: XX years

13. Intake of Calcium per day: XX mg/d

14. Intake of Vitamin D per day: XX IU/d

Physician: XXX

Nurse: XXX
